# Mycosporine-Like Amino Acids Promote Wound Healing through Focal Adhesion Kinase (FAK) and Mitogen-Activated Protein Kinases (MAP Kinases) Signaling Pathway in Keratinocytes

**DOI:** 10.3390/md13127056

**Published:** 2015-11-26

**Authors:** Yun-Hee Choi, Dong Joo Yang, Atul Kulkarni, Sang Hyun Moh, Ki Woo Kim

**Affiliations:** 1 Anti-aging Research Institute of BIO-FD & C Co. Ltd., Incheon 406-840, Korea; imyunhee@gmail.com (Y.-H.C.); atulkin@gmail.com (A.K.); 2 Departments of Pharmacology and Global Medical Science, Wonju College of Medicine, Yonsei University, Wonju 220-701, Korea; ydj1991@yonsei.ac.kr; 3 Institute of Lifestyle Medicine and Nuclear Receptor Research Consortium, Wonju College of Medicine, Yonsei University, Wonju 220-701, Korea

**Keywords:** Mycosporine-like amino acids (MAAs), wound healing, mitogen-activated protein (MAP) kinases, extracellular signal-regulated kinases (ERK), c-Jun N-terminal kinases (JNK)

## Abstract

Mycosporine-like amino acids (MAAs) are secondary metabolites found in diverse marine, freshwater, and terrestrial organisms. Evidence suggests that MAAs have several beneficial effects on skin homeostasis such as protection against UV radiation and reactive oxygen species (ROS). In addition, MAAs are also involved in the modulation of skin fibroblasts proliferation. However, the regulatory function of MAAs on wound repair in human skin is not yet clearly elucidated. To investigate the roles of MAAs on the wound healing process in human keratinocytes, three MAAs, Shinorine (SH), Mycosporine-glycine (M-Gly), and Porphyra (P334) were purified from *Chlamydomonas hedlyei* and *Porphyra yezoensis*. We found that SH, M-Gly, and P334 have significant effects on the wound healing process in human keratinocytes and these effects were mediated by activation of focal adhesion kinases (FAK), extracellular signal-regulated kinases (ERK), and c-Jun N-terminal kinases (JNK). These results suggest that MAAs accelerate wound repair by activating the FAK-MAPK signaling pathways. This study also indicates that MAAs can act as a new wound healing agent and further suggests that MAAs might be a novel biomaterial for wound healing therapies.

## 1. Introduction

In recent years, natural products from marine organisms have gained increasing research interest and potential economic importance as a number of novel compounds have been reported from different marine organisms [1,2,3,4]. Mycosporine-like amino acids (MAAs) are natural compounds found in a wide variety of organisms, including fungi, bacteria, cyanobacteria, phytoplankton, and macro-algae. MAAs are water-soluble, small with a molecular weight less than 400 Da, and colorless. More than 20 different MAAs have been identified in various organisms [5,6]. MAAs have received much attention for their functional roles in UV photo protection and ROS scavenging as they have been shown to diminish the direct and indirect damaging effects of environmental ultraviolet radiation (UVR) [7,8]. MAAs have also been shown to be highly resistant against abiotic stressors such as temperature, various solvents, and pH [5,6,7,9]. The increasing number of reports on the presence of MAAs in different marine species refers mostly to their potential photo protective properties [10]. Besides, it has been suggested that mycosporines and MAAs also have a role in osmotic regulation, particularly in cyanobacterial communities in hyper saline environments [9]. Further, MAAs have been reported to have antioxidant activity [11], and play regulatory roles in algal reproduction [12]. In addition, owing to the positive effects on cell regeneration observed in human skin fibroblasts, MAAs seem to be potential cosmeceutical agents [13,14,15]. In contrast with the abundant literature available on the functional roles of MAAs on the skin, there are no previous reports elucidating their beneficial effects on the wound healing process.

As the largest organ in the human body, the skin has several functions with the most important one being protection against various harmful stressors. The keratinized stratified epidermis and an underlying thick layer of collagen-rich dermal connective tissues are important components of the skin [16]. The outer covering of skin, the epidermis, is exposed to the environment and susceptible to injuries, therefore, the epidermis initiates the wound healing process in response to skin damage. Healing of skin wounds is a dynamic process involving highly integrated and overlapping stages: (1) the inflammatory stage consists of the extravasation of blood constituents, blood coagulation, and migration of immune cells to the wound site, (2) the proliferative phase involves the migration and proliferation of keratinocytes, fibroblast, and endothelial cells leading to re-epithelialization; and (3) the tissue remodeling phase restores tissue structural integrity and functional competence [17,18].

To facilitate proper wound healing and tissue repair, various factors such as cytokines and growth hormones play a major role [13,19]. In addition, tight regulation and activation of several signaling proteins involved in wound healing play crucial roles in the repair process. In this study, we examined the potential roles of three MAAs, mycosporine-glycine (M-Gly), shinorine (SH), and porphyra (P334) in wound healing. Further, we investigated the potential molecular mechanisms underlying the MAAs-mediated wound healing process.

## 2. Results

### 2.1. Characterization of MAAs

**Figure 1:**
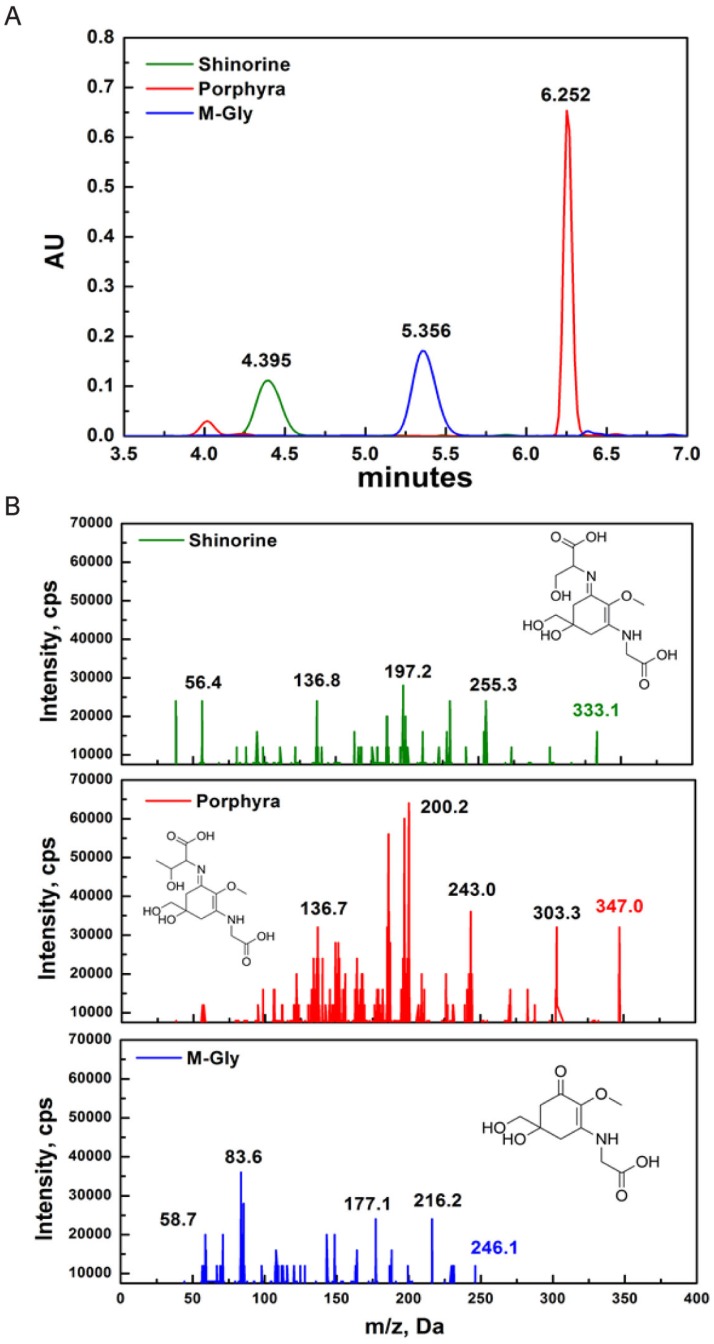
Characterization of MAAs (mycosporine-like amino acids): (**A**) HPLC-DAD (330 nm) chromatograms of SH, P334, and M-Gly. The retention time for SH, M-Gly, and P334 was 4.395, 5.356, and 6.252 minutes, respectively; (**B**) Triplequadruple ESI-MS/MS spectra of SH, P334, and M-Gly. Protonated parent molecule is indicated in bold type for each MAA (inset structure of respective MAA).

MAAs were identified based on their retention time during high-resolution reverse-phase liquid chromatography (HPLC) and their UV absorption spectra obtained from diode array detection (DAD). The retention time of SH, M-Gly and P334 was 4.395, 5.356, and 6.252 min, respectively (Figure 1A). Although DAD allows for fast acquisition of UV-VIS absorption spectra, it is difficult to finely distinguish each MAA compound based only on the absorption spectra because some MAAs have identical wavelength absorption maxima or only a few nm apart. To overcome this, we adopted high-resolution reverse-phase liquid chromatography/mass spectrometry (HPLC/MS) detection method to identify the specific MAAs. The positive electrospray ionization (ESI) mass spectral fragmentation patterns of SH, M-Gly, and P334 exhibited distinct characteristic features (Figure 1B).

### 2.2. Effect of MAAs on Cell Viability and Wound Healing

**Figure 2:**
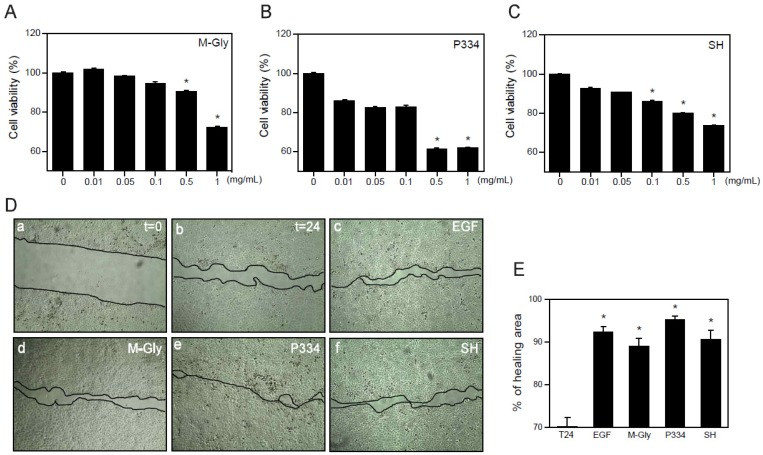
Wound healing effects of MAAs: (**A**–**C**) HaCaT cells were treated with indicated concentrations of M-Gly (**A**); P334 (**B**); or SH (**C**) for 24 h and cell viability was assessed by MTT assay; (**D**) HaCaT cells were scratched using pipet tips and incubated for 24 h in the presence of vehicle (*t* = 24, b), EGF (100 ng/mL, c), M-Gly (0.1 mg/mL, d), P334 (0.05 mg/mL, e), and SH (0.05 mg/mL, f). The *t* = 0 (a) indicates the time for scratching; (**E**) Percent (%) of healing area. Values are mean ± standard error of the mean (SEM). (* *p* < 0.05, one-way ANOVA followed by Bonferroni’s *post hoc* test for A to C and Student’s *t*-test for E).

Although many beneficial effects of MAAs such as UV protection and ROS scavenging have been suggested, the wound healing effects of MAAs on keratinocytes have not yet been reported. Before investigating the wound healing effects of MAAs on human keratinocytes (HaCaT), we analyzed the cytotoxicity of MAAs. Cells were treated with M-Gly, P334, and SH dose-dependently for 24 h and subjected to 3-(4,5-dimethylthiazol-2-yl)-2,5-diphenyltetrazoliumbromide (MTT) assay. Relatively high concentrations (0.5~1 mg/mL) of M-Gly and P334 treatment showed a significant increase in cytotoxicity but the cells were stable at lower concentrations ranging from 0.01 mg/mL to 0.1 mg/mL (Figure 2A,B). Cytotoxicity of SH was also minimal at low doses (0.01~0.05 mg/mL) (Figure 2C). These results indicate that low doses of M-Gly, P334, and SH have no cytotoxic effects on HaCaT cells.

The wound healing process is a dynamic procedure that includes inflammation, new tissue formation and remodeling [17]. Migration of keratinocytes is essential for wound healing and recovery from injury [20]. To investigate whether the M-Gly, P334, and SH play any role in the regulation of wound healing in human keratinocytes, we treated HaCaT cells with each MAA with the viable doses and examined the effects of MAAs in wound repair. M-Gly, P334 and SH significantly increased wound healing, exhibiting comparable potency to epidermal growth factor (EGF), a well-known wound healing factor (Figure 2D,E) [21]. These results indicate that these MAAs stimulate the wound repair process in human keratinocytes.

### 2.3. MAAs Induce the Wound Healing Process through Activation of FAK-MAP Kinases

**Figure 3:**
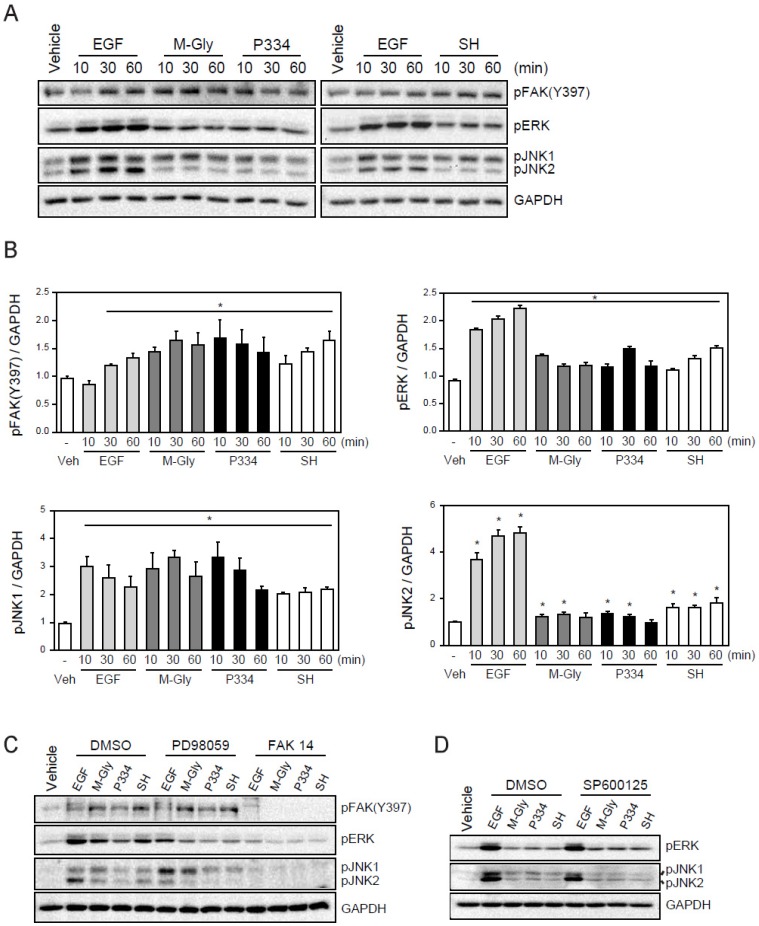
Activation of FAK (focal adhesion kinases), ERK(extracellular signal-regulated kinases), and JNK (c-Jun N-terminal kinases) by MAAs: (**A**) Activation of FAK, ERK, and JNK by treatment of EGF (100 ng/mL), M-Gly (0.1 mg/mL), and P334 (0.05 mg/mL), and SH (0.05 mg/mL) in HaCaT cells; (**B**) The intensity of each band was measured with densitometer and expressed as protein level normalized to GAPDH; (**C**) Effect of ERK (PD98059; 50 μM) and FAK (FAK14; 5 μM) inhibitors; (**D**) Effect of SP600125 (10 μM), a JNK inhibitor. GAPDH was used as an internal control. Values represent mean ± SEM (* *p* < 0.05, Student’s *t*-test).

We next sought to understand the underlying molecular mechanisms of wound healing facilitated by M-Gly, P334, and SH. A number of intracellular organizations and covalent modifications of signaling proteins act in concert to have an overall impact on the intricate process of cell migration. Focal adhesion kinase (FAK), a non-receptor protein tyrosine kinase (PTK) has been suggested as a critical regulator of cell migration. Indeed, FAK activation accelerates cell migration and wound healing [22]. Therefore, we examined if the MAAs-mediated wound repair resulted from FAK activation (Figure 2D,E). Treatment of M-Gly, P334, and SH significantly enhanced the phosphorylation of FAK at Y397 (Figure 3A,B). In addition, cells incubated with MAAs also showed ERK activation supporting the notion that the phosphorylation of Y397 in FAK can facilitate MAPKs extracellular signal-regulated kinase (ERK) activation (Figure 3A,B) [23]. The activation of FAK, ERK and JNK1/2 by MAAs were shown quantitatively in Figure 3A. These results indicate that the activation of FAK and ERK could be a mechanism by which MAAs induce wound healing in skin cells (Figure 3A,B). Furthermore, JNK has been shown to be required for *Drosophila* dorsal closure and is also known to play a role in cell migration [24,25,26,27]. In addition, recent studies demonstrated that JNK1 is required for the movement of fish keratinocytes and rat bladder tumor epithelial cells, possibly suggesting that the activation of JNK1 is involved in wound repair [28]. Thus, we examined whether the MAAs we tested could also activate JNK. Our results showed that treatment of MAAs induced the activation of JNK, especially JNK1, indicating that the wound healing effects by the three MAAs might be through, at least in part, the activation of JNK1 (Figure 3A). The activation of FAK, ERK, and JNK by the MAAs were specific because pre-treatment of inhibitors such as PD98059 (an ERK inhibitor), FAK14 (FAK inhibitor), or SP600125 (JNK inhibitor) significantly blunted their activation (Figure 3B,C). Inhibition of FAK also blocked the activation of ERK and JNK1, highly suggesting that the activation of FAK might be an upstream factor for ERK and JNK1 activation (Figure 3B).

### 2.4. Activation of ERK and JNK is Important for MAAs-Mediated Wound Healing

**Figure 4:**
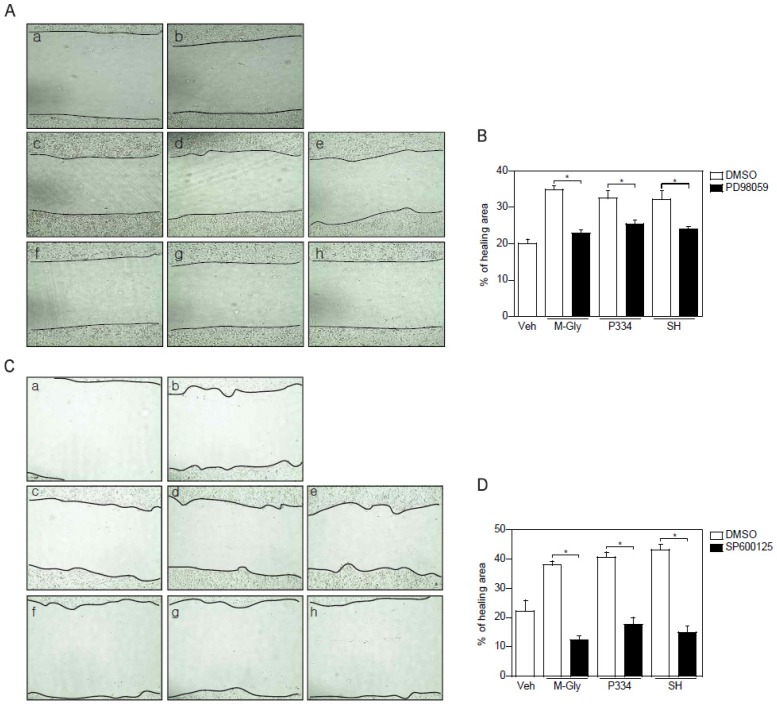
Inhibition of ERK and JNK impaired the MAAs-mediated wound healing effects. (**A**–**B**) Wound healing effects mediated by indicated MAAs was blunted by pretreatment of ERK inhibitor (PD98059; 50 μM). Scratched cells (a; *t* = 0) were incubated with DMSO (b), M-Gly (c), P334 (d), or SH (e) for 24 h. ERK inhibitor was pretreated for 2 h prior to incubation with M-Gly (f), P334 (g), or SH (h); (**C**–**D**) Wound healing effects mediated by indicated MAAs were decreased by pretreatment of JNK inhibitor (SP600125; 10 μM). Wounded cells (a; *t* = 0) were treated with DMSO (b), M-Gly (c), P334 (d), or SH (e) for 24 h. JNK inhibitor was pretreated for 2 h prior to incubation with M-Gly (f), P334 (g), or SH (h). The area of the wound was measured and the percentage of healed area calculated. Values represent mean ± SEM (* *p* < 0.05, Student’s *t*-test).

To verify the importance of the activation of ERK and JNK in MAAs-induced wound repair in skin cells, keratinocytes were pretreated with ERK and JNK inhibitors and subjected to wound healing assay. ERK inhibition using PD98059 showed reduction of wound healing by up to 15% (Figure 4A,B). However, inhibition of JNK completely blocked MAAs-mediated wound closure (Figure 4C,D). Altogether, these results indicate that acceleration of wound healing process by MAAs is mediated by activation of both ERK and JNK, supporting the notion that JNK might be the major player in MAAs-mediated wound healing in keratinocytes.

## 3. Discussion

Re-epithelialization occurs during wound healing as a result of keratinocyte migration into the wound site and this process is critical for proper healing of the wound. Impaired wound healing leads to major health complications such as predisposition to infections, long-term morbidity, skin ulcers and increased mortality [29]. MAAs are natural compounds found in a wide variety of marine and freshwater organisms, including fungi, bacteria, cyanobacteria, phytoplankton and macro-algae. So far up to 20 MAAs have been identified [5]. Beneficial effects of MAAs have been elucidated including UV-protective function, antioxidant role against ROS [11], and osmotic regulation [30]. However, the possible involvement of MAAs in the regulation of wound repair in skin cells has not yet been established. Here we explored the functional involvement of MAAs in the wound healing process in human keratinocytes. In addition, we investigated the underlying mechanisms mediating the MAAs’ effect in the wound repair process.

Activation of FAK has been known to play a major role in the wound healing process by regulating various processes such as cell proliferation and migration leading to re-epithelialization and tissue granulation during wound healing [31]. Moreover, FAK is a known scaffold protein for growth factor-induced migration and also regulates focal adhesion turnover [32]. For instance, a reduction in focal adhesion dissociation in FAK null cells leads to an increase in focal adhesion size and numbers [23]. This reduction in focal adhesion disassembly in FAK null cells can be rescued by constitutively active MEK, suggesting that ERK acts as a downstream effector on FAK-mediated focal adhesion dissociation [23]. In line with this, ERK, together with JNK, is known to be directly involved in converting extracellular signals into various cellular responses including proliferation, differentiation, and migration [33]. Interestingly, we found that MAAs strongly activated FAK. In addition, pharmacological inhibition of FAK reduced the activation of ERK and JNK, indicating that FAK is an upstream factor in MAAs-induced cell migration. Inhibition of ERK or JNK exhibited significantly blunted wound closure, supporting the notion that FAK and downstream factors such as ERK and JNK play important roles in the MAAs-mediated wound healing (Figure 4).

Activation of ERK by FAK in turn phosphorylates FAK at S910, which promotes the disassembly of focal adhesion (hemidesmosome disruption) during cell migration [34,35,36]. This hemidesmosome disruption mediated by FAK will facilitate phosphorylation of integrin β4 and consequently promote cell migration [37]. As shown in Figure 3, the MAAs we tested significantly elevated phosphorylation of FAK implying that the activation of FAK plays an essential role in the MAAs-mediated wound repair in keratinocytes. However, whether MAAs are directly involved in the hemidesmosome disruption or not warrants further studies.

JNK is generally thought to be a factor involved in inflammation, proliferation, and apoptosis [38]. In addition JNK has been known to be essential for cell migration and keratinocyte movement through phosphorylation of paxillin [28]. In agreement with this, our data showed that the activation of JNK is involved in the MAAs-induced cell migration. Inhibition of JNK using SP600125 completely blocked the MAAs-mediated wound healing suggesting that the JNK activation is crucial for the MAAs wound healing action (Figure 4C,D).

MAAs have been reported as potential ROS scavengers [39]. It is therefore possible that the wound healing effects of MAAs might be as a result of their role in balancing reactive oxygen species in the wound. Unbalanced or excessive ROS generation in the wound is deleterious for wound healing, though large amounts of ROS at a wound site are essential for protection against invading microorganisms or bacteria [40,41,42]. NADPH oxidase is one of the enzymes involved in regulating free radicals in cells and its activation leads to the production of highly reactive superoxide anion radicals [43,44]. Therefore, it will be interesting in the future to examine whether MAAs exert their positive effects on wound healing by regulating the reactive oxygen homeostasis through modulation of NADPH oxidase.

Effects such as antioxidant activity and UV-protective activity have evoked a substantial interest in MAAs as a beneficial material for human health. Our data indicate that the three MAAs we studied have wound healing potential. In particular, they activated FAK, ERK, and JNK, which play critical roles in cell proliferation, migration, and wound healing. Taken together, previous reports and our results strongly indicate the various potential roles of MAAs not only as cosmetics to protect the skin from harmful sun rays but also as drugs to facilitate the wound healing process. Furthermore, this knowledge on MAAs will likely result in the identification of novel targets for the treatment of wound healing disorders.

## 4. Material and Methods

### 4.1. Isolation and Analysis of MAAs

Three MAAs, porphyra-334 (P334), shinorine (SH), and mycosporine-gly (M-Gly), were extracted from *Chlamydomonas hedleyi* and *Porhyra yezoensis* that were obtained from the Korea Institute of Ocean Science and Technology, Korea. They were grown in an erlenmeyer flask (500 mL volume) containing 250 mL of Guillard’s f/2 medium (Sigma-Aldrich, St. Louis, MO, USA) with an initial cell density of 5 × 10^4^ cells/mL and suspended in a thermoregulated aquarium. A pH of between 8 and 9 was maintained in the medium by sparging CO_2_ (1%)-enriched air throughout the culture. After cells were grown for 7 days, 0.25 L of culture medium was harvested for dry weight biomass (a freeze-dryer was used for drying). In the culture system, dry weight (DW) and crude MAAs accounted for ~3.72 g/L and ~0.01 mg/g DW, respectively. The detailed protocol for MAAs isolation was described previously [45,46].

### 4.2. Characterization of MAAs

Dried algae (20 mg DW) were extracted for 2 h in screw-capped centrifuge vials filled with 20% aqueous methanol (*v*/*v*) at 45 °C. After 10 min centrifugation with 5000× *g* at room temperature, the supernatant was discarded and dried using vacuum at 45 °C (Jouan evaporator centrifuge RC 10.09, Cedex, France). The precipitate was dissolved in 500 μL of distilled water followed by the addition of 100 μL chloroform with gentle vortexing. After centrifugation for 5 min at 10,000× *g*, the upper water phase was transferred carefully into new microcentrifuge tubes to remove contaminating lipophilic photosynthetic pigments from water-soluble MAAs. Finally, the solvent was evaporated and the sample was dissolved in 50% methanol for further analysis.

All samples were analyzed quantitatively by HPLC (Shimadzu-LC20A, Seattle, WA, USA) with a DAD-SPD M20A detector (Quantum Northwest, Seattle, WA, USA), as described in a previous report [45]. Aliquots (30–60 μL) of the extract were diluted in 100% HPLC-grade methanol to a final volume of 0.5 mL and dried in a rotary evaporator. The residues were re-dissolved in 600 μL of water and then diluted (1:10) with aqueous trifluoroacetic acid 0.2% (pH 3.0) and ammonium hydroxide. The final solution was ultra-filtered with a Whatman 100 kDa filter (12,000× *g*, 20 min) to remove water-insoluble materials and large molecules.10 μL samples of the resulting solution were then injected into the HPLC system at a 1 mL/min flow rate. The signals were processed with the Class-VP software (Quantum Northwest, Seattle, WA, USA). Identification of MAAs (purity was 99.5%) was accomplished by their absorption maxima and retention times, calibrated with authenticated standards of shinorine, mycosporine-2-glycine, prophyra-334, which were provided by F. Figueroa, University of Malaga (Malaga, Spain). We ran the HPLC/MS of each MAA separately.

### 4.3. MS/MS Analysis

Dried MAAs extracts were dissolved in deionized water (1 mg/mL); a final concentration of 100 ppm was achieved using 50% methanol with 0.1% formic acid and passing it through a 0.45-μm membrane filter. Analyses of MAAs were performed on an ESI-MS/MS system (Thermo Fisher Scientific, San Jose, CA, USA) consisting of an AB SCIEX 3200 QTRAP MS/MS (Applied Biosystems, Foster City, CA, USA) with an ESI source (TurbolonSpray, Applied Biosystem/MDS SCIEX, Concord, Canada). Data acquisition and processing were performed with the AB SCIEX Analyst 1.5 software (AB SCIEX Korea Limited Company, Seoul, Korea). All MAAs were quantified in Product Ion (MS2) mode in positive mode. The syringe pump method properties (Tune Control) were as follows: syringe diameter 4.6 mm and flow rate 10.00 μL/min. The optimal ESI source conditions were as follows: turbo heater temperature (TEM) 300 °C, ion spray voltage 5500 V, curtain gas 10 psi, nebulizing gas (gas 1) 15 psi and heated gas (gas 2) 40 psi. The collision energy (CE) and entrance potential (EP) were set separately at 35 V and 4.50 V; the mass transition of MAAs, optimal declustering potential (DP) 51 V and collision cell exit potential (CXP) 4 V.

### 4.4. Cell Line

The human keratinocyte cell line, HaCaT, was purchased from the American Type Culture Collection (ATCC, Manassas, VA, USA). Cells were cultured in Dulbecco’s modified Eagle’s medium (DMEM, Hyclone, Logan, UT, USA) containing 10% heat-inactivated fetal bovine serum (FBS) and 2 mM L-glutamine and 100 U·m^−1^ penicillin-streptomycin in a humidified atmosphere [47].

### 4.5. MTT Assay

MTT assay (3-(4,5-dimethylthiazol-2-yl)-2,5-diphenyltetrazoliumbromide, Sigma-Aldrich, St. Louis, MO, USA) was used to determine cell viability. HaCaT cells (ATCC) were cultured in 96-well microplates and incubated in DMEM media containing 10% FBS. After 24 h, the cells were treated with the indicated doses of M-Gly, P334, and SH, and incubated for an additional 24 h. Afterwards, the media was replaced with 100 μL of MTT solution (0.5 mg/mL in cell culture medium) and incubated for 4 h at 37 °C. The MTT solution was then removed, and MTT formazan dissolved in 100 μL dimethyl sulfoxide (DMSO). Absorbance was measured at 540 nm using spectrophotometry.

### 4.6. Wound Healing Analysis

Keratinocytes were plated in six-well dishes and cultured to 100% confluence at 37 °C in 5% CO_2_. The tip of a 100 μL pipet was used to generate a scratch wound by manually scraping the cell surface [48,49]. The cells were washed once and cultured in 2 mL media with vehicle, EGF (Sigma-Aldrich, St. Louis, MO, USA), or MAAs (M-Gly, P334, or SH). An AxioObserver FL microscope (Advanced Microscopy Group, Bothell, WA, USA) was used to acquire the cell images at ×10 magnification at the indicated time points. The cell images of the wounded region were acquired from more than three locations. The percentage of the wound closure was calculated using *ImageJ* software (http://imagej.nih.gov/ij/index.html). All the experiments were done in triplicate.

### 4.7. Western Blot Analysis

Cells were washed with PBS 1× and lysed with RIPA buffer (150 mM NaCl, 50 nM Tris, 1% Triton-X-100, 0.5% sodium deoxycholate and 0.1% SDS) containing protease and phosphatase inhibitors (Roche). Extracts were isolated by centrifugation at 12,000× *g* for 1 min. The protein concentration was measured using Bio-Rad Protein Assay reagent (Bio-Rad Laboratories, Hercules, CA, USA). Equal amounts of protein were loaded and electrophoresis done on SDS–polyacrylamide gels, and then transferred to nitrocellulose membranes. The membranes were blocked in 5% skim milk in Tris-buffered saline containing 0.1% Tween 20 for 1 h at room temperature. The blocked membranes were then incubated with primary antibodies overnight at 4 °C with agitation followed by incubation with horseradish peroxidase-conjugated secondary antibodies for 1 h at room temperature. The blots were visualized using the Chemiluminescence Western Blot Detection System (BioSpectrum^®^ 600 Imaging System, Upland, CA, USA). Primary antibodies used were as follows: pFAK (pY397, BD biosciences, San Jose, CA, USA), pErk (Cell Signaling, Danvers, MA, USA), pAkt (Cell Signaling, Danvers, MA, USA), pJNK (Cell Signaling, Danvers, MA, USA), and GAPDH (Santa Cruz Biotechnology, Santa Cruz, CA, USA). Inhibitors used were as follows: PD98059 (an ERK inhibitor, Cell signaling, Danvers, MA, USA), FAK14 (FAK inhibitor, Santa Cruz Biotechnology, CA, USA), or SP600125 (JNK inhibitor, Santa Cruz Biotechnology, Santa Cruz, CA, USA).

### 4.8. Statistical Analysis

All experiments were performed in at least three independent experimental replicates. Data are presented as the mean and standard error of the mean. Statistical significance was determined using either one-way ANOVA followed by Bonferroni’s *post hoc* test or two-tailed Student’s *t*-test. *p* < 0.05 was considered as statistical significance.
